# Transcriptome Analysis of the *Sm*-Mediated Hypersensitive Response to *Stemphylium lycopersici* in Tomato

**DOI:** 10.3389/fpls.2017.01257

**Published:** 2017-07-19

**Authors:** Huanhuan Yang, Tingting Zhao, Jingbin Jiang, Xiuling Chen, He Zhang, Guan Liu, Dongye Zhang, Chong Du, Songbo Wang, Xiangyang Xu, Jingfu Li

**Affiliations:** ^1^College of Horticulture, Northeast Agricultural University Harbin, China; ^2^Beijing Genomics Institute Shenzhen, China

**Keywords:** *S. lycopersici*, *Sm* tomato, RNA-seq, regulatory resistance mechanisms, differentially expressed genes

## Abstract

Gray leaf spot disease caused by *Stemphylium lycopersici* is a major disease in cultivated tomato plants and threatens tomato-growing areas worldwide. *Sm* is a single dominant gene that confers resistance to tomato gray leaf spot disease agent. However, the underlying molecular mechanism remains unclear. Here, resistant (cv. Motelle, containing the *Sm* gene) and susceptible (cv. Moneymaker) plants were inoculated with virulent *Stemphylium lycopersici* isolate at a time point at which both cultivars showed a strong response to *S. lycopersici* infection. Transcriptome analyses were performed in both cultivars using RNA-seq. The number of differentially expressed genes (DEGs) was higher in Motelle than Moneymaker. Functional classification revealed that most DEGs were involved in plant–pathogen interactions, plant hormone signal transduction, regulation of autophagy, glycerophospholipid metabolism, and α-linolenic acid metabolism. Moreover, the genes that were significantly up-regulated in *Sm* tomatoes were involved in plant–pathogen interaction pathways. A total of 26 genes were selected for confirmation of differentially expressed levels by quantitative real-time PCR. This knowledge will yield new insights into the molecular mechanism of *Sm* responses to *S. lycopersici* infection.

## Introduction

Gray leaf spot disease is considered one of the most devastating diseases in plants such as pepper (Cho et al., 2001), cotton (Francovig et al., 1999), and spinach (Koike et al., 2001). Tomato gray leaf spot disease is caused by three species of *Stemphylium*: *Stemphylium solani*, *Stemphylium floridanum*, and *Stemphylium* *lycopersici* (Miranda et al., 2010). Gray leaf spot disease is considered a major disease in cultivated tomatoes and has threatened tomato-growing areas worldwide (Simmons, 2001). In the early stages, tomato gray leaf spot disease symptoms appear as brownish-black specks, which later expand to necrotic lesions with gray centers and dark brown borders. As the disease progresses, affected leaves became chlorotic, with perforated centers of lesions, ultimately leading to leaf drying and falling. *S. lycopersici* has been established as a cause tomato gray leaf spot disease based on morphology and molecular identification (Graham and Zeiders, 1960).

However, resistance is often governed by a ‘gene-for-gene’ interaction (Dangl and Jones, 2001), in which plants carrying a resistance (R) gene specifically recognize a pathogen carrying a corresponding avirulence (avr) gene. The avr gene is recognized by an effector protein after this protein is secreted into the apoplastic space during infection (Nekrasov et al., 2006), which induces either a compatible or incompatible interaction between the fungus and infected plant. An incompatible interaction (chlorosis) results in rapid cell death at the site of infection, which is called the hypersensitive response (HR), whereas a compatible interaction occurs when the pathogen can grow and ramify, causing necrosis in the infected cells (Hammond-Kosack and Jones, 1996; Soylu et al., 2003; Soylu et al., 2004; Pei et al., 2012). To date, only one tomato gray leaf spot disease resistance gene, the dominant gene *Sm*, has been identified and maps to chromosome 11 near two markers, TG110 and T10, in the tomato genome (Behare et al., 1991). *Sm* was derived from the wild tomato species *S. pimpinellifolium*, which was used to breed resistant tomato cultivars (Dennett, 1950). To the best of our knowledge, little is known about the mechanisms of tomato gray leaf spot disease resistance. Therefore, a comprehensive transcriptome analysis will provide a valuable resource for understanding tomato gray leaf spot disease resistance mechanisms.

With the development of second-generation sequencing technology, RNA-seq has become a useful tool for the comprehensive analysis of host–pathogen interactions in plants (Varshney et al., 2009; Haas and Zody, 2010; Du et al., 2014) such as wheat (Yang et al., 2015), rice (Bai et al., 2015), maize (Li et al., 2010), cabbage (Wang et al., 2016), cucumber (Zhang et al., 2014), and eggplant (Yang et al., 2017). Notably, the present study is the first to use Illumina RNA-seq to analyze the regulatory resistance mechanisms of the *Sm* tomato cultivar in response to *S. lycopersici*. This study may provide a basis for cloning *Sm* resistance genes, which will be useful for understanding the regulatory mechanisms involved in plant–pathogen interactions.

## Materials and Methods

### Plant Materials and *S. lycopersici* Inoculation

Two tomato cultivars, the resistant cv. Motelle containing the *Sm* gene (kindly provided by the Asian Vegetable Research and Development Center, AVRDC) and the susceptible cv. Moneymaker (kindly provided by the Chinese Academy of Agricultural Sciences), were used in this study. *S. lycopersici* was plated on potato dextrose agar (PDA) in Petri dishes. The isolated pathogen was incubated at 28°C for 5–10 days with a 12-h photoperiod. Tomato seedlings were sprayed with a conidial suspension (1 × 10^4^ conidia/ml). Mock-treated plants were sprayed with sterilized water. All plants were maintained in a greenhouse at 28°C with relative humidity >85% (Sun et al., 2016).

### Microscopy

To identify the interaction process of *Sm*-mediated HR and key time points involved in the mechanism, we used lactophenol trypan blue staining and scanning electron microscopy to provide a basis for the RNA-seq and RT-qPCR analyses (Franco et al., 2008; Zhao et al., 2015; Wang et al., 2016). Leaf samples of the resistant and susceptible cultivars were sampled at 0, 1, 2, 3, 4, 5, 6, 7, and 8 days after inoculation and observed under a light microscope. Moreover, the inoculated samples were cut into pieces of approximately 2 mm × 5 mm, soaked for 1.5 h in 2.5% glutaraldehyde (pH 6.8) at 4°C, and rinsed three times in 0.1 M phosphate buffer (pH 6.8) followed by a 50, 70, 90, and 100% ethanol dehydration series. The leaves were then soaked in 100% ethanol:tert-butyl alcohol at a 1:1 ratio followed by 100% tert-butyl alcohol. The leaves were placed in a refrigerator at -20°C for 30 min and then placed in a freeze-dryer (Hitachi ES-2030, Japan). Finally, scanning electron microscope (Hitachi S-3400, Japan) was used to observe the progress of Sm-mediated HR.

### RNA Extraction and Illumina Sequencing

Total leaf RNA was collected at the 0 time point (mock-treatment, including resistant and susceptible cultivars), 5 days after incompatible interaction (resistant post-inoculation, RPI) and 5 days after compatible interaction (susceptible post-inoculation, SPI). Total RNA was extracted from three biological replicates for each treatment with three plants according to an RNeasy Plant Mini Kit extraction protocol and was then used in the RT-qPCR experiments (Schroeder et al., 2006; Fang et al., 2015). Total RNA was treated with DNase I, and oligo (dT) was used to isolate mRNA. After addition of the fragmentation buffer, mRNAs were fragmented. Then, cDNA was synthesized using the mRNA fragments as templates. The suitable fragments were selected for PCR amplification. RNA-seq library preparation and sequencing were performed by BGI Tech (Shenzhen, China). The libraries were generated using the NEBNext^®^ Ultra^TM^ RNA Library Prep Kit for Illumina^®^ (NEB, United States). Then, the library was sequenced using an Illumina HiSeq 4000, and 150-bp paired-end reads were generated.

### Illumina Reads and Differentially Expressed Genes (DEGs)

SOAPnuke software^1^ was used to filter reads. Primary sequencing data (called raw reads) were cleaned by removing reads with adapters. A low-quality read was defined based on the percentage of bases in a read with a quality less than 15 or greater than 20%. Low-quality reads (sequencing quality less than 5) were also removed.

Clean reads were identified by filtering low-quality data and mapped to the *S. lycopersicum* reference genome sequence (Tomato Genome Consortium, 2012). Gene expression levels in terms of transcripts were quantified by RSEM (RNA-seq by expectation maximization) and FPKM (fragment per kilobase per million mapped; Trapnell et al., 2010; Li and Dewey, 2011). HISAT was used to align paired-end clean reads to the reference genome (Kim et al., 2015). Differentially expressed genes (DEGs) were detected using NOIseq methods with a Noisy Distribution Model (Anders and Huber, 2010; Tarazona et al., 2015) and are shown using a Venn diagram. Genes with a divergence probability (*P*_NOI_) ≥ 0.8 and log_2_ fold-change ≥ 2 were defined as significantly enriched (Benjamini and Hochberg, 1995). Novel transcripts were reconstructed using StringTie (Pertea et al., 2015).

### Gene Ontology and KEGG Pathway Analysis of DEGs

The GO seq R package was used for Gene Ontology (GO) analysis of DEGs, and GO terms with an adjusted *P*-value < 0.05 were considered significantly enriched in DEGs (Chen et al., 2005). KOBAS was used for KEGG metabolic pathway analysis, and *P*-values ≤ 0.05 were defined as significantly enriched (Kanehisa et al., 2010).

### Quantitative Real-Time PCR Analysis

A total of 26 DEGs were analyzed by RT-qPCR to verify the expression profiles obtained by RNA-seq. Reverse transcription was performed using the Reverse Transcriptase M-MLV (RNase H-) reverse transcription kit (TaKaRa) according to the manufacturer’s instructions. Data analysis was performed using the 2^ΔΔCT^ method (Livak and Schmittgen, 2001) with EFa1 (R: 5′-CCACCAATCTTGTACACATCC-3′, S: 5′-AGACCACCAAGTACTACTGCAC-3′) as a reference gene for normalization (Supplementary Table S2).

## Results

### Microscopic Analysis of *S. lycopersici* Invasion in the Two Tomato Cultivars

**FIGURE 1:**
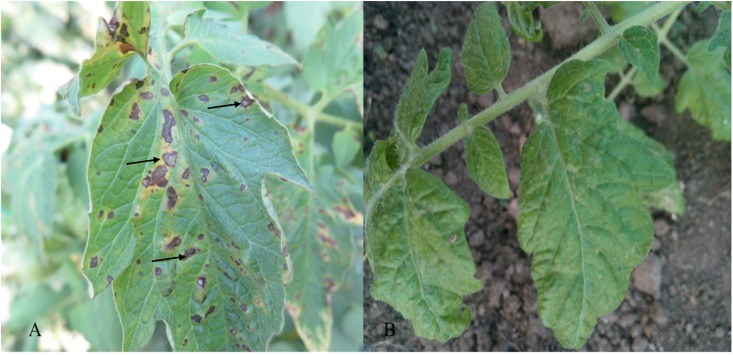
Typical disease symptoms observed as necrotic leaf spot (arrows) on leaves of susceptible cv. Moneymaker **(A)** and lack of any visible symptoms on resistant cv Motelle **(B)** 5 days after inoculation.

After 3–5 days, symptoms of gray leaf spot disease appeared on the tomatoes. **Figure 1** shows the tomatoes 5 days after inoculation. Plants carrying *Sm* resistance gene displayed strong HR post-inoculation as shown in **Figure 1B**, whereas susceptible plants had perforated centers of the lesions at 5 days after inoculation (**Figure 1A**).

**FIGURE 2:**
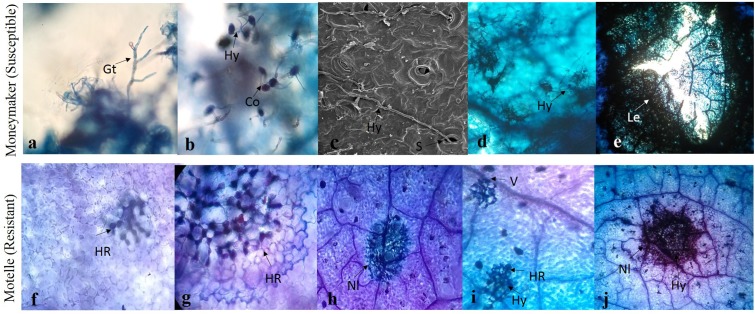
Trypan blue stained-tomato leaf samples inoculated with *S. lycopersici.* The *S. lycopersici* infection process **(a–e)** in Moneymaker (Susceptible). Germ tube extension were shown in **(a)**. Conidiophores germinated and hypha growth at 2 or 3 dpi **(b)**, the hypha invaded into stomata at 3 days after inoculation in Moneymaker and Sm tomato **(c)**. The hypha continued to invade and expand in Moneymaker **(d)**. As disease progressed, affected leaves expanded to necrotic lesions and the centers of lesions became perforated **(e)**. The hypersensitive cell death response **(f–j)** in Motelle (Resistant). The hypersensitive-like symptom was found at 4 days after inoculation in resistant tomato **(f)**. At 5 days after inoculation, the cell wall of resistant tomato were formed **(g)**. Hyphal growth was restricted in the necrotic lesions on resistant tomato at 6 days after inoculation **(h)**. The increasing necrotic lesions were showed in mesophyll cells and leaf veins at 7–8 days after inoculation **(i,j)**. Hy, hyphae; S, stomata; Nl, Necrotic lesions; V, leaf veins; HR, hypersensitive response.

To identify the interaction process involved in *Sm*-mediated HR and the key time points for this mechanism, lactophenol trypan blue staining and scanning electron microscopy were performed. Symptoms and different phenotypical responses triggered by *S. lycopersici* in resistant cv. Motelle (incompatible interaction) and susceptible cv. Moneymaker (compatible interaction) were observed. The microscopic analysis showed that germ tube extension occurred at 1–2 days (**Figure 2**). Conidiophore germination and hypha growth occurred at 2 or 3 days, and the hypha invaded the stomata at 3 days after inoculation in cv. Moneymaker. No difference was observed between Motelle and Moneymaker at 3 days after inoculation. In the compatible interaction, the hypha continued to invade and expand in cv. Moneymaker (Thomma et al., 2005) as the disease progressed, the affected areas of the leaves expanded to form necrotic lesions, and the centers of lesions became perforated. Furthermore, HR was observed at 4 days after inoculation in Motelle. At 5 days after inoculation, the cell wall of Motelle formed. Hyphal growth was restricted to the necrotic lesions on Motelle at 6 days after inoculation. Increasing necrotic lesions were apparent in the mesophyll cells and leaf veins at 7–8 days after inoculation.

### Summary of RNA-seq Data

In this study, an average of ∼8.78 Gb were generated from each sample using the Illumina HiSeq platform. The raw data were deposited in the NCBI Sequence Read Archive under the accession number SRP097450. Ultimately, 37,657 novel transcripts were generated with 16,388 unknown splicing events for known genes, 3,775 novel coding transcripts without any known features, and 17,494 transcripts for long non-coding RNA. Illumina quality scores of 20 (Q20) and 30 (Q30) represent the percentages of sequencing data with error rates less than 1 and 0.1%, respectively (Cock et al., 2010). In this study, more than 99% of reads were ≥Q20, and 97% of these clean reads were ≥Q30. Only data with a quality score ≥Q30 were used for next analyses. After filtering, 53.7–62.1 million clean reads were generated, and at least 86% of these reads were mapped to the tomato reference genome (Supplementary Table S1); of these, more than 85% of the clean reads were uniquely mapped reads, and 0.89% were multiply mapped to tomato chromosomes.

### DEGs in Response to *S. lycopersici*

**FIGURE 3:**
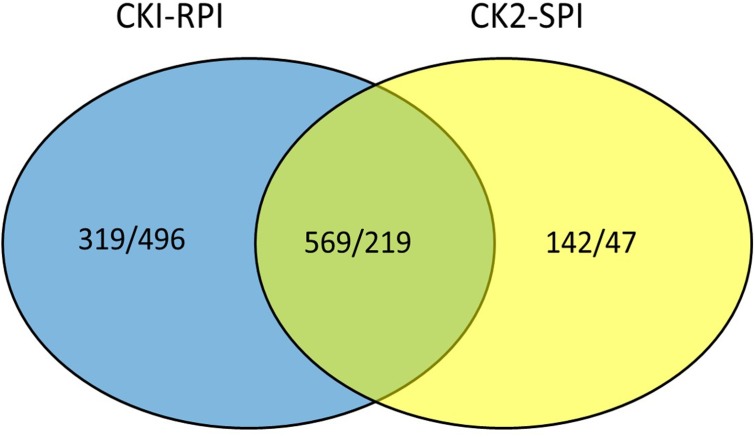
Venn diagram showing up/down-regulated in CK1-RPI and CK2-SPI post-inoculation *Stemphylium lycopersici.* CK1 and CK2: Resistant cultivar and susceptible cultivar were inoculated with water. RPI and SPI: Resistant cultivar and susceptible cultivar were inoculated with *Stemphylium lycopersici.*

A gene was considered significantly differentially expressed when *P*_NOI_ ≥ 0.8 and log_2_-fold ≥ 2. The two standards were used to identify DEGs in the R and S cultivars in response to *S. lycopersici* over 5 days after inoculation. All FPKM values for each gene and the fold-changes and *P*_NOI_ for DEGs are shown in Supplementary Tables S3, S4, respectively. Overall, the number of DEGs was significantly higher in RPI compared with SPI at 5 days after inoculation. Additionally, the number of up-regulated genes was greater than the number of down-regulated genes in the two tomato cultivars. Overall, 1,603 and 977 genes were differentially expressed in the R and S cultivars, respectively, of which 569 and 219 genes were up- and down-regulated, respectively (**Figure 3**).

### GO and KEGG Enrichment Analysis of DEGs

**FIGURE 4:**
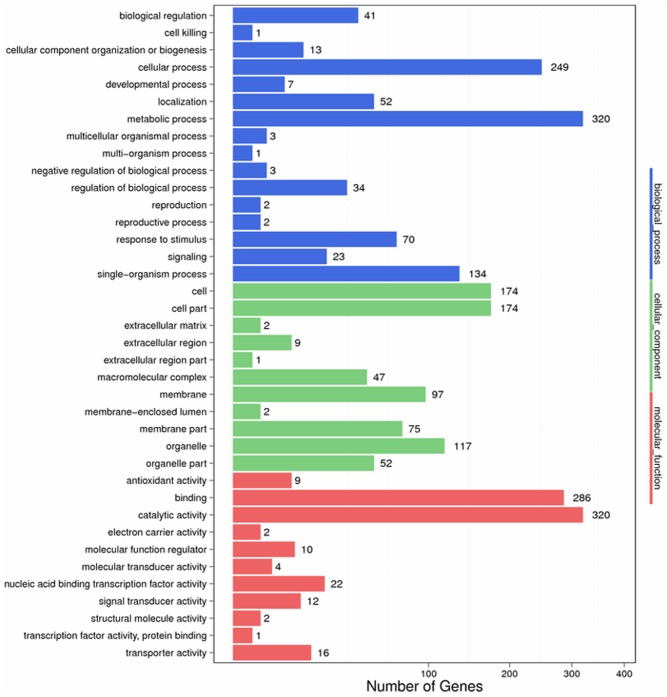
Gene ontology categories of differentially expressed genes (DEGs) in *Sm* tomato in response to *S. lycopersici* infection.

To identify the functions of DEGs involved in the response to *S. lycopersici*, we determined GO assignments by using GO seq (Young et al., 2010). Most of the assigned functions of DEGs belonged to the biological process, cellular component and molecular function categories. In the biological process category, significantly enriched terms were metabolic process, cellular process, single organism process, response to stimulus, biological regulation, regulation of biological process and signaling, and these terms were related to disease resistance. In the cellular component category, significantly enriched terms included cell, cell part, organelle part, and membrane, which were found to be specific to the resistant cultivar. In the molecular function category, significantly enriched terms included catalytic activity, binding, nucleic acid binding transcription factor activity, transporter activity, and signal and transducer activity. Additionally, binding and catalytic activity terms were found to play a critical role in plant hormone signal transduction (**Figure 4**).

To investigate the biological pathways associated with DEGs, all DEGs were subjected to KEGG pathway analysis. In the CK1-RPI group, DEGs were significantly enriched in six metabolic pathways with *Q*- and *P*-values < 0.05: “Plant–pathogen interaction” (111 DEGs), “Regulation of autophagy” (23 DEGs), “Plant hormone signal transduction” (78 DEGs), “Glycerophospholipid metabolism” (24 DEGs), “alpha-Linolenic acid metabolism” (15 DEGs), and “Glycerolipid metabolism” (21 DEGs) (**Table 1**). These categories are shown in a scatter plot of the KEGG pathway enrichment of DEGs.

**Table 1:** Significantly enriched KEGG pathway of differentially expressed genes (DEGs) in response to *S. lycopersici*.

	Pathway	Number of up-regulated genes	Number of down-regulated genes	Pathway ID
CK1-RPI	Plant–pathogen interaction	88	23	ko04626
	Regulation of autophagy	17	5	ko04140
	Plant hormone signal transduction	50	28	ko04075
	Glycerophospholipid metabolism	19	5	ko00564
	alpha-Linolenic acid metabolism	12	3	ko00592
	Glycerolipid metabolism	12	9	ko00561
CK2-SPI	Plant hormone signal transduction	34	13	ko04075
	Glycerophospholipid metabolism	15	2	ko00564
	Alpha-Linolenic acid metabolism	8	0	ko00592
	Glycerolipid metabolism	9	5	ko00561

**FIGURE 5:**
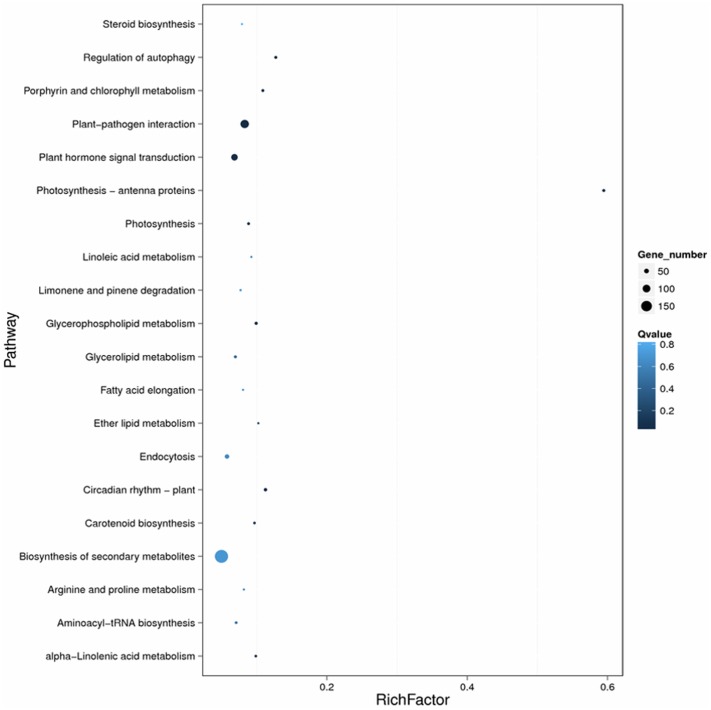
Scatter plot of the KEGG pathway enrichment of DEGs. Rich factor is the ratio of the DEG number to the background number in a certain pathway. The size of the dots represents the number of genes, and the color of the dots represents the range of the *q*-value.

The enrichment factor is the ratio of the DEG number to the background number in a certain pathway (**Figure 5**). As shown in **Figure 5**, the number of genes and the enrichment factor in the pathways “Plant–pathogen interaction,” “Regulation of autophagy,” “Plant hormone signal transduction,” and “Biosynthesis of secondary metabolism” were significantly higher than in the other pathways. Many other disease-resistance pathways, including Photosynthesis-antenna proteins, Photosynthesis, Circadian rhythm-plant, Porphyrin and chlorophyll metabolism, Carotenoid biosynthesis, Ether lipid metabolism, and Aminoacyl-tRNA biosynthesis, were also enriched. In total, the most-enriched pathways, ‘Plant–pathogen interaction (111 DEGs)’ and ‘Plant hormone signal transduction (78 DEGs) (**Tables 2**, **3**), may be the major metabolic pathways involved in the *Sm* tomato response to *S. lycopersici* infection.

The size of the dots represents the number of genes, and the color of the dots represents the range of *q*-values. However, in the CK2-SPI group, DEGs were enriched for four major metabolic pathways with *P*-values < 0.05: “Plant hormone signal transduction” (47 DEGs), “Glycerophospholipid metabolism” (17 DEGs), “alpha-Linolenic acid metabolism” (8 DEGs), and “Glycerolipid metabolism” (14 DEGs). Therefore, DEGs related to disease-resistance pathways were significantly up-regulated in the *Sm* tomato cultivar at 5 days after inoculation with *S. lycopersici* (**Table 2**). Among the DEGs, 19 disease-resistance genes in the significantly enriched KEGG pathway “Plant–pathogen interaction” exhibited significant differences in expression after RPI compared with their expression after SPI in response to *S. lycopersici* infection in the *Sm* tomato cultivar and Moneymaker at 5 days post-inoculation. The network analysis of “Plant–pathogen interaction” predicted a response to *S. lycopersici* infection (**Table 2**). Finally, 20 DEGs in the significantly enriched KEGG pathway “Plant hormone signal transduction” were identified (**Table 3**).

**Table 2:** Differentially expressed genes in the significantly enriched KEGG pathway “Plant–pathogen interaction” in tomato R cultivar and S cultivar at 5 days after inoculation (RPI, SPI).

		*P*_1_			*P*_2_		
		RPKM		log_2_ Fold-change	RPKM		log_2_ Fold-change
Gene name	Homologous protein in KEGG	CK1	RPI	CK1-RPI	CK2	SPI	CK2-SPI
Solyc07g055280.2.1	WRKY5	11.68	61.08	2.39	15.36	28.33	0.88
Solyc09g072810.2.1	LRR	3.03	64.95	4.42	6.60	65.87	3.32
Solyc04g072070.2.1	WRKY51	4.95	40.62	3.04	16.33	102.25	2.65
Solyc02g072190.2.1	WRKY65	2.58	21.61	3.07	4.52	14.10	1.64
Solyc04g079420.2.1	PR	24.04	98.43	2.03	51.49	57.50	0.16
Solyc03g123860.2.1	RLPK	0.01	67.13	12.71	0.01	40.53	11.98
Solyc08g016210.2.1	LRR	1.09	57.44	5.72	6.59	45.84	2.80
Solyc02g071130.2.1	WRKY71	1.06	18.91	4.16	5.89	19.34	1.71
Solyc03g093610.1.1	ETH	40.47	499.24	3.62	25.61	368.30	3.85
Solyc02g070890.2.1	LRR	4.04	59.75	3.89	3.60	45.68	3.67
Solyc07g053170.2.1	MAPK	35.49	149.79	2.08	43.95	104.31	1.25
Solyc06g070990.2.1	WRKY61	0.36	33.30	6.52	0.56	18.38	5.04
Solyc11g072660.1.1	PSPK	6.06	41.87	2.79	6.55	29.21	2.16
Solyc08g016310.2.1	LRR	6.18	217.71	5.14	12.38	172.23	3.80
Solyc04g078420.1.1	MYB	93.39	378.77	2.02	102.33	287.94	1.49
Solyc02g077370.1.1	PTI5	6.67	75.95	3.51	19.71	183.77	3.22
Solyc01g067010.2.1	KRP	27.78	6.07	-2.20	15.61	6.48	-1.27
Solyc05g012890.1.1	PLBR	2.58	17.46	2.76	3.17	28.65	3.17
Solyc09g083050.2.1	COA	58.55	6.56	-3.16	29.79	5.42	-2.46

**Table 3:** Differentially expressed genes in the significantly enriched KEGG pathway “Plant hormone signal transduction” in tomato R cultivar and S cultivar at 5 days after inoculation (RPI, SPI).

		Fold change (log_2_ ratio)	
Gene name	Annotations	CK1-VS-RPI	CK2-VS-SPI
Solyc10g085310.1.1	Abscisic acid receptor PYR/PYL family	2.90	1.45
Solyc02g065470.1.1	Pathogenesis-related protein 1	2.01	1.14
Solyc03g082520.1.1	SAUR family protein	3.56	4.12
Solyc07g062980.2.1	Protein brassinosteroid insensitive	-3.48	-2.64
Solyc03g082530.1.1	SAUR family protein	3.88	4.38
Solyc09g089930.1.1	Ethylene-responsive transcription factor 1	5.30	3.01
Solyc01g008910.2.1	DELLA protein	-4.31	-2.62
Solyc06g050500.2.1	Abscisic acid receptor PYR/PYL family	3.18	2.69
Solyc02g073580.1.1	Transcription factor TGA	2.63	6.07
Solyc01g103050.2.1	Auxin response factor	2.93	1.97
Solyc04g076970.2.1	Transcription factor TGA	3.16	1.31
Solyc08g036660.2.1	Jasmonate ZIM domain-containing protein	5.07	3.78
Solyc02g085340.1.1	DELLA protein	-3.17	-2.32
Solyc02g069310.2.1	Regulatory protein NPR1	1.98	1.05
Solyc03g114210.2.1	Serine/threonine-protein kinase CTR1	1.87	0.45
Solyc08g016310.2.1	Kinesin family member C2/C3	5.14	3.80
Solyc03g122190.2.1	Jasmonate ZIM domain-containing protein	3.65	2.89
Solyc02g077370.1.1	Ethylene-responsive transcription factor	3.51	3.22
Solyc09g065850.2.1	Auxin-responsive protein IAA	3.43	4.74
Solyc08g079140.1.1	SAUR family protein	9.96	4.40

### Validation of RNA-seq Data by RT-qPCR

To verify the expression profiles obtained from RNA-seq and predict the defense-response process, we analyzed 26 DEGs [WRKY transcription factor, PR1 protein precursor, disease-resistance protein, receptor-like protein kinase, leucine-rich repeat (LRR) receptor-like serine/threonine-protein kinase, pathogenesis-related gene transcriptional activator, ethylene-responsive transcription factor, jasmonate ZIM domain-containing protein, and abscisic acid receptor PYL9] in the Plant–pathogen interaction, Regulation of autophagy, Plant hormone signal transduction, Glycerophospholipid metabolism, alpha-Linolenic acid metabolism, and Glycerolipid metabolism pathways using RT-qPCR (Supplementary Table S2). A significant positive correlation between the RT-qPCR results and RNA-seq data was detected, indicating that the RNA-seq data were reliable with a strong positive correlation coefficient (*R*^2^ = 0.9491).

## Discussion

*Sm* is considered an effective gene for resistance to tomato gray leaf spot disease caused by *S. lycopersici.* In the present study, RNA-seq was used to verify the transcriptome profiles of *Sm* tomato in response to *S. lycopersici* infection. The reliability of the RNA-seq dataset was verified by the significant positive correlation between the RT-qPCR results and RNA-seq data. Ultimately, many significant DEGs were identified between the R and S cultivars in response to *S. lycopersici* infection. The overall number of DEGs was significantly higher in *Sm* tomatoes compared with cv. Moneymaker at 5 days after inoculation, and the number of up- and down-regulated genes in the *Sm* tomato cultivar was higher than that in cv. Moneymaker. Significantly enriched GO terms in the biological process category included metabolic process, cellular process, single response to stimulus, biological regulation, regulation of biological process, and signaling. Moreover, these terms were related to disease resistance. A total of 17 up-regulated genes in the plant–pathogen interaction pathway were analyzed in the R and S cultivars at 5 days after inoculation. Previous studies and functional annotations of genes showed that these up-regulated genes were related to defense responses against fungi (Van Loon et al., 2006).

Previous studies have indicated that the pathogenesis-related (PR) gene transcriptional activator PTI5 belongs to a specific family of defense-related proteins involved in defense against pathogens in plants (Jones and Dangl, 2006). These findings demonstrated a positive role of PTI5 in the regulation of defense genes and disease resistance, suggesting that a pathogen-activated post-transcriptional regulatory process is necessary for the pathogen-mediated induction of defense gene expression. Similar results were obtained in our study: Solyc02 g077370.1.1 (PR gene transcriptional activator PTI5) was shown to be involved in the enriched KEGG pathway “Plant–pathogen interaction,” and the up-regulated expression levels suggested that PTI5-type proteins in tomato may play specific roles in the response to *S. lycopersici.*

Plant hormones are known to regulate the expression of gene networks related to defense responses (Bari and Jones, 2009), among which jasmonic acid (JA), salicylic acid (SA), and ethylene (ET) play vital roles in resistance to biotrophic and necrotrophic pathogens, such as stress responses, oxide-reduction processes, and cell wall and wax biosynthesis processes (Grant and Lamb, 2006; Van Loon et al., 2006). Additionally, activation of signaling, such as by SA, JA, and ET, will induce defense responses that include most PR proteins. Previous studies have shown that ET responses are vital for *B. cinerea* resistance in tomato leaves. As Fu et al. (2014) demonstrated, ET and SA play important roles in the defense response of tomato against *V. dahlia*. In our study, based on KEGG analysis, 20 DEGs were identified in the significantly enriched KEGG pathway “Plant hormone signal transduction.” These results are consistent with previous studies (Shamrai, 2014) that showed that plant hormones and other defense-related proteins are involved in disease resistance. Interestingly, ERF1 (ethylene-responsive transcription factor1), JAZ1 (jasmonate ZIM domain-containing protein), and SAUR family proteins were identified in the KEGG pathway “Plant hormone signal transduction” in the present study, suggesting that JA, ET, and SAUR family proteins may play roles in the resistance of *Sm* tomato to *S. lycopersici*. Similarly, previous studies have shown that ERF transcription factors were the connecting factors of signal cross-linking pathway, which were involved in a variety of plant hormone signaling pathways and play an important role in resistance to biotic and abiotic stresses (Gutterson and Reuber, 2004).

**FIGURE 6:**
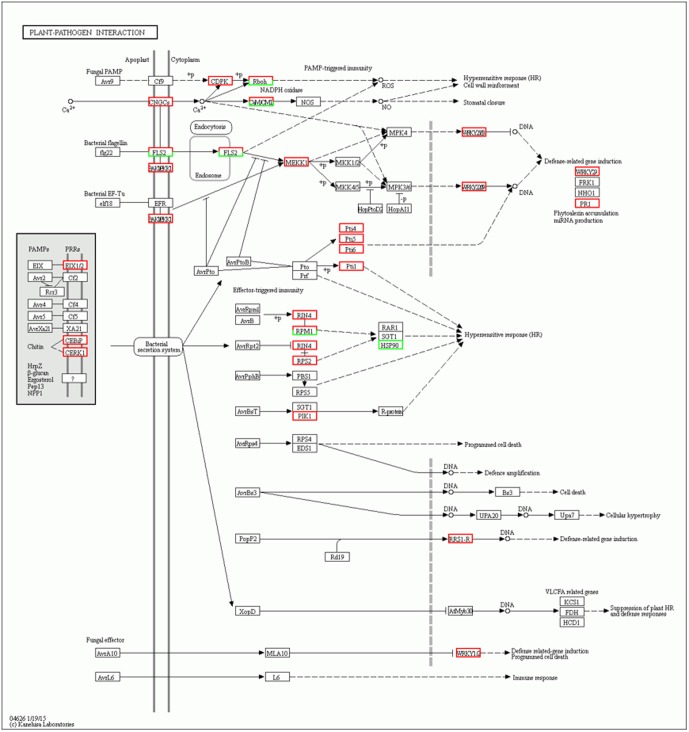
Network analysis of “Plant–pathogen interaction” predicts a response to *Stemphylium lycopersici* infection.

Intracellular Ca^2+^ influx is considered a key and early event downstream of multiple pathogen-associated molecular pattern (PAMP) sensing, resulting in local and systemic acquired resistance (Lecourieux et al., 2005; Boudsocq et al., 2010). Accordingly, calcium-dependent protein kinase (CDPK) is immediately induced by the interaction of flg22 with Avr-Cf9 (Romeis et al., 2000). In addition, recent advances have identified CDPKs as central regulators of Ca^2+^-mediated immune and stress responses that are crucial signaling nodes mediating plant responses to both abiotic stress and pathogens (Boudsocq and Sheen, 2013). Interestingly, the results of our study showed that six CDPK genes (Solyc02g083850.2.1, Solyc10g050060.1.1, Solyc01g107740.2.1, Solyc10g079130.1.1, Solyc03g033540.2.1, and Solyc03g113390.2.1) were induced at 5 days after inoculation in the *Sm* tomato cultivar. Based on the results, we therefore propose that these CDPK genes are involved in JA- and SA-mediated defense responses of tomato against *S. lycopersici* (**Figure 6**). Similarly, Hu et al. (2016) demonstrated that CDPK genes play critical roles in plant responses to both abiotic stress and pathogens.

**FIGURE 7:**
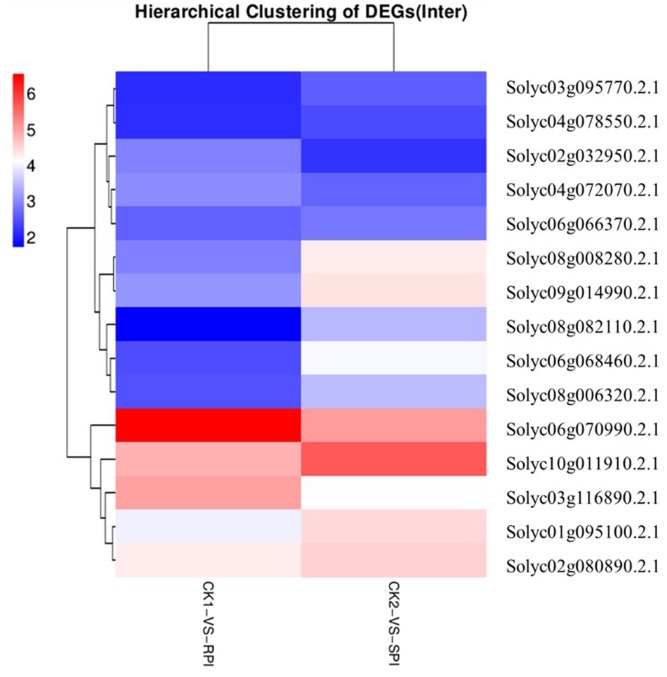
Differentially expressed WRKY genes in RPI and SPI.

The recognition of PAMPs initiates downstream signaling pathways involving WRKY transcription factors to promote defense responses against bacterial and fungal pathogens and nematodes (Asai et al., 2002; Bhattarai et al., 2010). In this study, based on KEGG analysis, a total of 15 WRKY genes were differentially expressed as shown by the hierarchical clustering of DEGs in both tomato cultivars (**Figure 7**). Among them, 5 WRKY genes, Solyc07g055280.2.1, Solyc04g072070.2.1, Solyc02g072190.2.1, Solyc02g071130.2.1, and Solyc06g070990.2.1, which have been associated with defense responses against pathogens in previous studies (Spyropoulou et al., 2014; Du et al., 2015), were specifically up-regulated in the *Sm* tomato cultivar. In our study, these findings were also validated by RT-qPCR analysis, suggesting that WRKY transcription factors may play a role in the resistance of tomato *Sm* to *S. lycopersici*, similar to previous studies. These results suggest that the five WRKY genes may activate a series of downstream PR genes and play a key role in defense responses of tomato to *S. lycopersici.*

Furthermore, a series of PR proteins was activated at this stage. PR proteins are known to be induced upon pathogen invasion and can restrict pathogen growth (Van Loon et al., 2006). PR-1 proteins are a highly conserved family of plant proteins; however, the mechanism of PR-1 protein induction by pathogens has not been elucidated. PR-1 was recently shown to have antifungal activity (Stintzi et al., 1993). In our study, 19 DEGs in the significantly enriched KEGG pathway “Plant–pathogen interaction” were significantly up-regulated in the *Sm* tomato cultivar after inoculation. Solyc04 g079420.2.1, a resistance-related gene, was up-regulated. The results obtained from our study suggest the enrichment of KEGG pathway “Plant–pathogen interaction” and the higher expression levels indicate that the PR-1 type proteins of tomato may play significant roles in the response to *S. lycopersici.*

**FIGURE 8:**
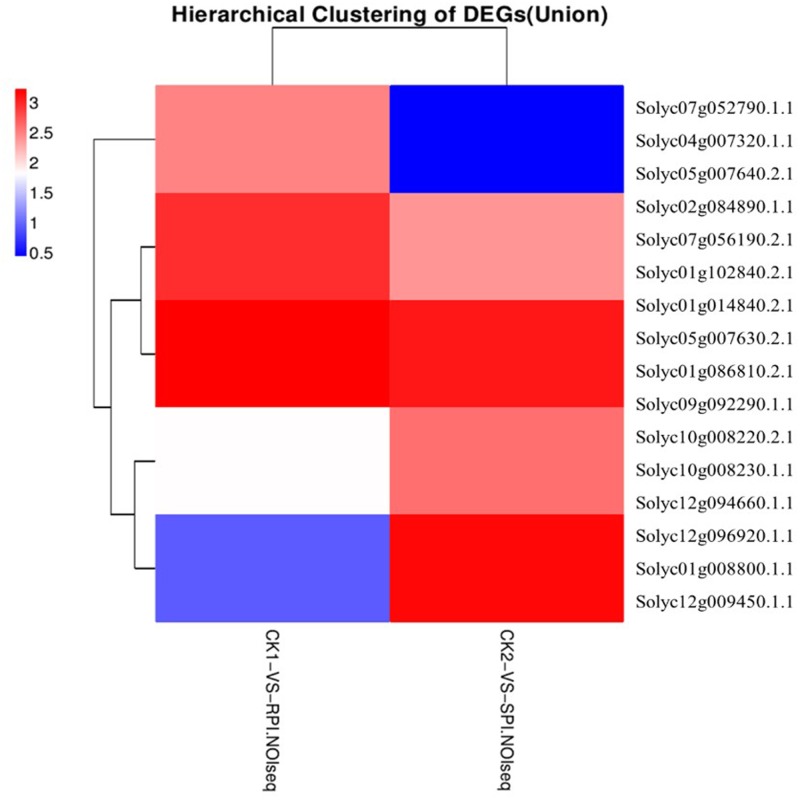
Differentially expressed R genes in RPI and SPI.

**FIGURE 9:**
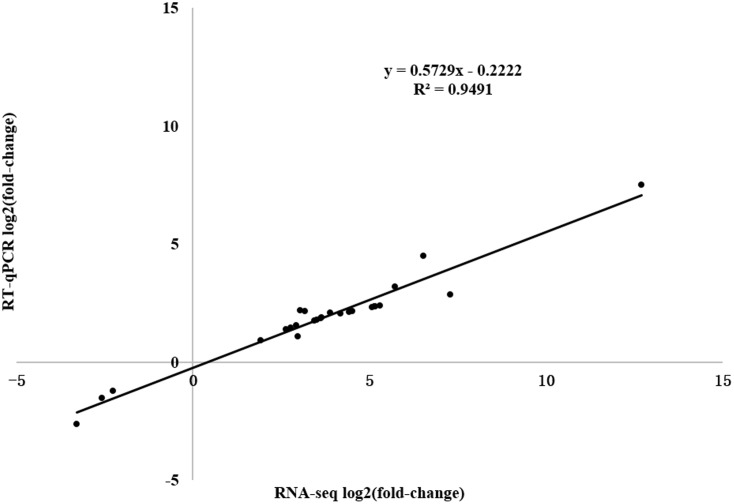
Correlation of expression levels between RNA-Seq and RT-qPCR in *Sm* tomato.

Plant disease resistance is generally dominated by the gene-for-gene hypothesis, which states that the AVR-encoding gene product of a pathogen is specifically recognized, either directly or indirectly, by plant disease R gene products. The R gene encodes a putative NBS-LRR class member that contains a nucleotide-binding site (NBS) and a carboxyl-terminal LRR (Vander Biezen and Jones, 1998). During plant–pathogen interactions, previous studies indicated that specific disease R proteins are generally related to downstream signaling transduction associated with pathogen resistance and are activated by specific effectors induced by R gene expression levels (De Young and Innes, 2006). Accordingly, additional efforts should be focused on putative R genes, including the NB-LRR domain, which may be induced by *S. lycopersici* infection. In our study, the number of up-regulated putative R genes was greater in the *Sm* tomato cultivar than in the tomato S cultivar. A total of 15 R genes were differentially expressed based on the hierarchical clustering of DEGs in both tomato cultivars (**Figure 8**). Moreover, two putative R genes encoding the TMV resistance N-like protein (Solyc07g052790.1.1 and Solyc04g007320.1.1) and RPM1 (Solyc05g007640.2.1 and Solyc05g007630.2.1) were specifically up-regulated (log_2_ fold change ≥ 2) in the tomato *Sm* cultivar at 5 days after inoculation. These results were validated by RT-qPCR analysis (**Figure 9**). Mackey et al. (2002) demonstrated that RPM1 is an NBS-LRR protein from *Arabidopsis thaliana* that confers resistance to *Pseudomonas syringae* expressing either *avrRpm1* or *avrB*. RPM1 is also a peripheral membrane protein that likely resides on the cytoplasmic surface of the plasma membrane and is related to the onset of the HR (Mackey et al., 2002; Soylu et al., 2005). Consistent with these previous studies, the expression levels of these putative R genes were higher in the tomato *Sm* cultivar compared with cv. Moneymaker. These genes may play critical roles in the *Sm* tomato response to *S. lycopersici* infection and may be candidate genes induced by *S. lycopersici* infection.

When a specific part of the plant is infected by a pathogen, systemic acquired resistance is activated, and several downstream defense genes (such as PR1) and plant antitoxin may also be activated, inducing HR to prevent pathogen growth by cell death. HR is a common fungal defense response in tomato. In contrast to field resistance, HR is generally controlled by single dominant genes. *Sm* (which is found in the resistant cv. Motelle) is a dominant gene for resistance to tomato gray leaf spot disease caused by *S. lycopersici* (Behare et al., 1991). Morphological changes and microscopic observations were used to identify the interaction process in Motelle. The morphological changes that occurred in the interaction in Motelle, which showed a strong HR upon inoculation, at 5 days post-inoculation are shown in **Figure 1B**. By contrast, cv. Moneymaker exhibited perforated lesion centers (**Figure 1A**). These observations are consistent with our microscopic observation studies, which revealed no difference between Motelle and Moneymaker at 3 days after inoculation. The basal defense response of resistant genes was activated at this stage. Additionally, co-regulated genes in both resistant and susceptible tomato were shown to promote expression levels and increase the resistance response to *S. lycopersici* infection. Furthermore, HR-like symptoms were observed at 4 days after inoculation in Motelle. At 5 days after inoculation, the cell wall of Motelle formed, indicating a strong HR. To our knowledge, this report is the first to demonstrate the interaction process involved in Motelle.

In summary, microscopic and RNA-seq analyses were performed to observe interactions between *S. lycopersici* and *Sm* tomatoes. Microscopic analyses revealed hypersensitive-like symptoms at 4 days after inoculation in the resistant cv. Motelle plant at site of interactions. Network analysis was performed to identify *S. lycopersici*-responsive regulatory pathways. As the mycelium of *S. lycopersici* invades the stomata and mesophyll cells, some effector proteins secreted by *S. lycopersici* are rapidly recognized by the *Sm* tomato. This triggers downstream defense signaling transduction associated with the Ca^2+^ channel and several other pathways, including those involving JA, SA, and ET (ERF1). Subsequently, specific defense-related transcription factors, such as WRKY proteins (Moore et al., 2011; Van Verk et al., 2011; Puranik et al., 2012), are triggered, which activates the R genes and regulates a series of downstream resistance pathways. Finally, HR is induced, causing the death of cells surrounding the infection sites and limiting pathogen growth. These results suggest the potential mechanism of *Sm* tomato against *S. lycopersici* infection.

### Database Link and Accessions

The clean data of all samples have been submitted to NCBI^2^ (Kodama et al., 2012), and each SRA accession corresponding to the treatment name was listed in Supplementary Table S5. The accession numbers is: SRP097450.

## Author Contributions

HY, JL, XX, TZ, and JJ conceived and designed the experiments. XC, GL, DZ, CD, SW and HZ, performed the RNA isolation and qRT-PCR experiments. HY performed the data analysis and wrote the manuscript. All authors read and approved the final manuscript.

## Conflict of Interest Statement

The authors declare that the research was conducted in the absence of any commercial or financial relationships that could be construed as a potential conflict of interest.

## Footnotes

1 https://github.com/BGI-flexlab/SOAPnuke

2 http://www.ncbi.nlm.nih.gov/sra
