# Supplementary material for: Transcriptome Analysis of the Sm-Mediated Hypersensitive Response to Stemphylium lycopersici in Tomato
Source: Front Plant Sci. 2017 Jul 19;8:1257. doi: 10.3389/fpls.2017.01257 (PMC5515834; doi:10.3389/fpls.2017.01257)
Supplement: Supplementary file 1 [file Table_1.DOCX]

| Sample | Total Raw Reads(Mb) | Total Clean Reads(Mb) | Total Clean Bases(Gb) | Clean Reads Q20(%) | Clean Reads Q30(%) | Clean Reads Ratio(%) | Total CleanReads | Total MappingRatio | Uniquely MappingRatio |
| --- | --- | --- | --- | --- | --- | --- | --- | --- | --- |
| CK1 | 58.64 | 53.66 | 8.05 | 98.87 | 96.51 | 91.50 | 53656317 | 86 | 84 |
| CK2 | 68.11 | 62.13 | 9.32 | 98.88 | 96.55 | 91.19 | 62130614. | 87 | 85 |
| RPI | 68.53 | 61.04 | 9.16 | 98.87 | 96.50 | 89.14 | 61040389 | 86 | 84 |
| SPI | 65.09 | 58.94 | 8.84 | 98.87 | 96.52 | 90.61 | 58942440 | 86 | 85 |

TABLE S1 Summary of sequencing reads after filtering.
