# Supplementary material for: Transcriptome Analysis of the Sm-Mediated Hypersensitive Response to Stemphylium lycopersici in Tomato
Source: Front Plant Sci. 2017 Jul 19;8:1257. doi: 10.3389/fpls.2017.01257 (PMC5515834; doi:10.3389/fpls.2017.01257)
Supplement: Supplementary file 2 [file Table_2.DOCX]

Table S2 .Primers used for qPCR.

| Primer name | Forward primer sequence (5’-3’) | Reverse primer sequence (5’-3’) |
| --- | --- | --- |
| 08g081610 | AACAACAAGTGGTGGCTCCTA | CCCTTTGAACTGCTGCACCTA |
| 01g106620 | GGATGCAACACTCTGGTGGA | CGTTGTTGCACCTGACCCTA |
| 09g072810 | CTGCTGGATTGTGTAGCCCA | TGTTGCCTTCAAGCCGTACT |
| 04g072070 | ACCATGTTTGGCTCTTCCACT | GAACATGTGTTGGTGTGGCAT |
| 03g123860 | TGCTACTCTAGGCCAGCTCA | TGCAACTGGGTGAGTGATCC |
| 08g016210 | GACCTTCGCCGCAACAATTT | GCCTTTTCGAACGCATCCAG |
| 03g093610 | ACAGGGGCGTTAGACAAAGG | TCCCCGGCTCTCAACTTCTA |
| 02g070890 | GGGTTGGGGCAGTTATCCAA | GGTGGAATGGCACCTGAGAA |
| 06g070990 | TCATCCATCTCAGCATCCGC | GCTGCAATTGTGTCTGGTGG |
| 08g016310 | TCACTGGGGAGATTCCGAGA | GTTCCAGTCCACCACCCAAT |
| 02g077370 | GGCTAGACATGGTGCGAGAG | GCCTTAGCACCTCGCATTCT |
| 05g012890 | CTCGGCATTATGAGGTCGCT | AAGCCCGTCGCACTTTCTTA |
| 01g109880 | GCCGTAGGCTTGAGTCACTT | TGCAAGACATCGGCAGTCAT |
| 04g078840 | GTTTAGGAGCCAGTGGGGTC | CTGCCTCCTTTCAACGACCT |
| 05g015840 | CGAGACCAACGCTTCTGTCA | TGACCCGAAGAGTCAGGGAA |
| 09g089930 | TCCGAAACAGTCACATCGCA | AGCATCTTCCGCGCTATCAA |
| 08g082180 | TGTATCCGAGCGCTTAGCTG | CAATTCACGCACAGCCACAA |
| 06g050500 | AGCGGTTAAACAACCGCAAC | TTATCGAATCGGCGGACGAG |
| 02g073580 | CGTGGATCACAGGGTGGAAG | TCCTTAACCCTTTCAACGCCA |
| 01g103050 | TTGTCGCAGCAACACCTACA | TTGGGCAATCCAGCAACTCA |
| 08g036660 | AGAGCAGAAATCTGAGCCGTTA | TGTGTTCTTGTCACCACACG |
| 08g036620 | CCTAGCAACTTGTTGATGGAGG | TGATGAAGGCTCAGACAGCTT |
| 03g122190 | GTTGCACCACAGCAACAACA | GCTAATTTCGGCAGCCTGTG |
| 01g106630 | CGTTGTTGCACCTGACCCTA | GGATGCAACACTCTGGTGGA |
| 09g065850 | CTCCTGCACCAAAAGCACAA | CCAACAAGCATCCAATCACCA |
| 11g016930  EFa1 | ACAAGGCTCAAGGTGCTCTC  CCACCAATCTTGTACACATCC | GGCTAATGTTCCACCGCAGA  AGACCACCAAGTACTACTGCAC |
