# Supplementary material for: Transcriptome Analysis of the Sm-Mediated Hypersensitive Response to Stemphylium lycopersici in Tomato
Source: Front Plant Sci. 2017 Jul 19;8:1257. doi: 10.3389/fpls.2017.01257 (PMC5515834; doi:10.3389/fpls.2017.01257)
Supplement: Supplementary file 5 [file Table_5.DOCX]

Table S5. Each SRA accession corresponding to the treatment name

| Sample name | Accession | Treatment name |
| --- | --- | --- |
| SPI_3  SPI_2  SPI_1  RPI_3  RPI_2  RPI_1  CK2_3  CK2_2  CK2_1  CK1_3  CK1 2  CK1 1 | SRX2508623  SRX2508624  SRX2508625  SRX2508626  SRX2508627  SRX2508628  SRX2508629  SRX2508630  SRX2508631  SRX2508632  SRX2508633  SRX2508634 | Susceptible post-inoculation, SPI  Susceptible post-inoculation, SPI  Susceptible post-inoculation, SPI  Resistant post-inoculation, RPI  Resistant post-inoculation, RPI  Resistant post-inoculation, RPI  Susceptible mock-treatment, CK2  Susceptible mock-treatment, CK2  Susceptible mock-treatment, CK2  Resistant mock-treatment, CK1  Resistant mock-treatment, CK1  Resistant mock-treatment, CK1 |
